# Royal Jelly Improves the Morphology of the Reproductive Tract, Internal Egg Quality, and Blood Biochemical Parameters in Laying Hens at the Late Stage of Production

**DOI:** 10.3390/ani11071861

**Published:** 2021-06-23

**Authors:** Mahmoud S. El-Tarabany, Mohamed Abdo Nassan, Ayman S. Salah

**Affiliations:** 1 Department of Animal Wealth Development, Faculty of Veterinary Medicine, Zagazig University, P.O. Box 44511 Sharkia, Egypt; 2 Department of Clinical Laboratory Sciences, Turabah University College, Taif University, P.O. Box 11099, Taif 21944, Saudi Arabia; m.nassan@tu.edu.sa; 3 Department of Animal Nutrition and Clinical Nutrition, Faculty of Veterinary Medicine, New Valley University, P.O. Box 72511 El-Kharga, Egypt; asabry3999@yahoo.com

**Keywords:** laying hen, royal jelly, egg production, reproduction, animal welfare

## Abstract

**Simple Summary:**

The main targets of senescence in laying hens are the ovaries and mostly the follicles. Recently, there has been great attention on the investigation of beehive products, including royal jelly (RJ), for their various functional, biological, and pharmaceutical benefits. Therefore, the purpose of this study was to evaluate the impacts of RJ administration on the egg production rate, ovarian and follicular patterns, and blood chemistry of aged Lohman Brown laying hens. RJ treatment, either 100 or 200 mg kg^−1^ body weight, may improve the morphology of the reproductive tract (including follicular growth and oviduct morphology), egg production rate, and internal egg quality traits of aged laying hens.

**Abstract:**

The purpose of this study was to evaluate the effects of royal jelly (RJ) on the morphology of the reproductive tract, egg production, and blood biochemical indices of aged laying hens. In total, 120 Lohman Brown laying hens at week 58 of age were randomly assigned into three equal groups. Pure RJ was dissolved in distilled water and injected subcutaneously as follows: the first treatment (R_1_; 100 mg RJ kg^−1^); the second treatment (R_2_; 200 mg RJ kg^−1^); the control treatment (CON; 1 mL distilled water). Both RJ-treated groups exhibited a significantly higher number of large yellow follicles (LYFs), small yellow follicles (SYFs), and large white follicles (LWFs) (*p* ˂ 0.05). Furthermore, RJ treatment significantly increased the diameter and weight of the F1 follicles. However, only the R2 group exhibited significantly greater ovary and uterus weights. RJ treatment did not affect the percentage of oviduct and weight of ovarian stroma. In addition, RJ increased the hen–day egg production rate compared with the CON group; however, only the R_2_ group showed greater egg weight (*p* = 0.032). RJ treatment also improved the albumen height, Haugh units, and yolk index. The administration of RJ significantly decreased the serum glucose, but increased the levels of serum albumen and cholesterol. In conclusion, RJ treatment may improve the morphology of the reproductive tract (including follicular growth and oviduct morphology), egg production rate, and internal egg quality traits of aged laying hens.

## 1. Introduction

It is widely accepted that the reproductive ability of females deteriorates with age. Moreover, ovaries and their follicles are the most likely organs exposed to senescence in females [1]. In lab animals, the reduction of ovarian activity is initiated during midlife, with a gradual depletion of the follicular reserve [2]. Similarly, as in mammals, the reproductive indices of hens progressively decrease as age is increased, and the size of the clutch is reduced with an increment in the interval between ovulations. Another sign of impaired reproduction in aged hens is the increased frequency of anovulatory cycles and missed eggs within the clutch [3]. This naturally occurs at the end of the production cycle in laying hens [4].

Similar to a bunch of grapes in appearance, the ovary of a laying hen is a cluster of numerous follicles [5]. Ovarian follicles, however, are ordered in a size hierarchy ranging from 6 to 20 mm [6]. Comparing the rate of follicular maturation in young and aging hens, one can observe that the former is much slower [7]. Various factors are associated with the onset of the gradual decline in egg production in aged birds, which include the sizes of yolky follicles as well as the changes in the accumulation pattern of yolk material into the follicles and the progressive rate of atresia in the small follicles [8]. Additionally, more delicate changes in the levels of sex steroids, namely timing or amplitude of the pre-ovulatory surge of hormones, are believed to be responsible for the fluctuation in follicular growth of aged laying hens [9].

Recently, investigation of bee products has greatly increased (e.g., for products such as propolis, bee pollen, royal jelly (RJ), and bee venom), mainly due to their various biological functions and pharmaceutical benefits [10]. In this context, RJ is a yellowish-white honey bee secretion of creamy liquid structure and is mainly used for larvae and adult queen nourishment. Chemically, RJ is a rich source of proteins (12–16%), sugar (10–16%), lipids (3–6%), and vitamins [11]. Additionally, RJ is rich source of proteins and essential amino acids, and contains small quantities of a hormone-rich substance (testosterone). RJ has been determined to exhibit antioxidant, antimicrobial, and immunological activities [12], besides growth-promoting functions [13]. Therefore, the purpose of this study was to evaluate the impacts of RJ administration on egg production rate, ovarian and follicular patterns, and blood chemistry of aged Lohman Brown laying hens.

## 2. Materials and Methods

### 2.1. Experimental Design, Birds, and Management

A total of 120 Lohman Brown laying hens at week 58 of age were obtained from a commercial flock, where the average hen–day egg production (HDEP) was 77.5% and average body weight was 1720 ± 74 g. Birds were randomly assigned into three groups (40 birds). Each group comprised 10 replicate cages (4 birds/cage). The cage dimensions were 50 × 49 × 48 cm as L × W × H. Throughout the experimental period (6 weeks), birds were kept in an environmentally controlled house (24 ± 2 °C and 50–60% relative humidity). The light regimen was 16 h/day (0600 to 2200 h). All birds were fed a corn-soybean diet (Table 1).

The chemical composition of pure RJ (Pharco Company for pharmaceutical industries) was specified by gas chromatography–mass spectrometry analyses. The main flavonoid components are described in Table 2. On a weekly basis (6 consecutive weeks from 58 to 63 weeks of age), the pure RJ was prepared in distilled water and injected subcutaneously as follows: the first treatment (R1; 100 mg RJ kg^−1^); the second treatment (R2; 200 mg RJ kg^−1^); the control group (CON; 1 mL distilled water). The experimental design and the dose of RJ treatment were adjusted according to our previous trial, which was primarily conducted on commercial Tetra laying hens [14].

### 2.2. Egg Production and Internal Egg Quality

The daily collection of eggs was performed 3 times/day, and then the calculation of HDEP was performed as follows: the number of daily eggs produced/the number of birds alive. Egg weight was determined using an electronic balance (±0.01 g, 1202 MP, Sartorius, Germany). To estimate the internal egg quality, 240 fresh eggs (80 eggs from each group) were collected in 4 batches from week 59 to week 62 of age. Individually, each egg was labeled and the internal egg quality parameters were evaluated within 12 h. Gently, each egg was broken and the internal contents were evacuated onto a flat glass surface. The yolk and albumen were separated and weighed. In accordance with the method of Reddy et al. [15], the width and height of yolk and albumen (mm) were evaluated using a sensitive electronic caliper. The indices of yolk quality were determined according to the method of Romanoff and Romanoff [16], where yolk index (%) = yolk height (cm)/yolk diameter (cm) × 100. Haugh units (HU) were computed according to Haugh [17] as follows: HU = 100 log (H + 7.57 − 1.7W0.37), where “H” and “W” refer to albumin height (mm) and egg weight (g), respectively.

### 2.3. Blood Sampling and Biochemical Indices

Two blood samples (each 1.5 mL) were collected at weeks 60 and 62 of age (20 hens/group) by a brachial-vein puncture. In order to minimize the stress magnitude, the sampling process was finished within 2 min. The first blood sample was collected into EDTA tubes to evaluate the hematological parameters [18]. The second sample was collected into plain tubes to obtain serum by centrifusion (3000 rpm for 15 min). Thereafter, the blood chemistry was evaluated (total proteins, triglycerides, cholesterol, and albumen using Diamond (Diamond, 2345 N) commercial kits [19].

### 2.4. Morphology of Reproductive Tract

At the end of the experiment (the 63rd week of age), two birds from five replicates per group were randomly selected and slaughtered. Immediately after slaughtering, whole oviduct, ovary, and accompanying follicular hierarchy were removed. If there was an egg in the oviduct, it was removed and considered as a laid egg. Accordingly, former researchers concluded that the follicular size is a better criterion for the follicular maturity than the weight of the yolk-free mass [20]. The number of large yellow follicles (diameter > 10 mm; LYFs), small yellow follicles (diameter 5–10 mm; SYFs), and large white follicles (diameter 3–5 mm; LWFs) were calculated according to Renema et al. [21]. Furthermore, the weight and diameter of the largest yellow follicle (F1 follicle) were measured along and across the stigma (within ±0.01 mm) using sensitive calipers [22]. Weights and percentages were also estimated for the ovary, oviduct, uterus, and ovarian stroma (within ±0.01 g).

### 2.5. Statistical Analyses

All statistics were performed using SPSS software (Version 16.0; IBM Corp., Armonk, NY, USA). Kolmogorov–Smirnov tests were performed in order to confirm the normality of the data as well as the homogeneity of variances. As the variables are normally distributed, data were analyzed using the general linear model procedure (repeated measures analysis of variance). The mixed model comprised the fixed effect of the treatment (CON, R_1_ and R_2_) and the random effects (batches and error).

## 3. Results

Both RJ treated groups exhibited higher number of LYFs, SYFs, and LWFs (Table 3). Furthermore, RJ treatment significantly increased the diameter and weight of the F1 follicles. However, only the R_2_ group exhibited significantly greater ovary and uterus weights. On the contrary, the RJ treatment did not affect the percentage of oviduct and weight of ovarian stroma.

RJ treatment significantly increased the HDEP %. However, only the R_2_ group showed significantly higher egg weight (Table 4). Furthermore, the R_2_ group had a greater yolk ratio. The RJ treatment also improved the albumen height, Haugh units, and yolk index. On the contrary, the RJ treatment did not affect the albumen weight and ratio.

**Figure 1:**
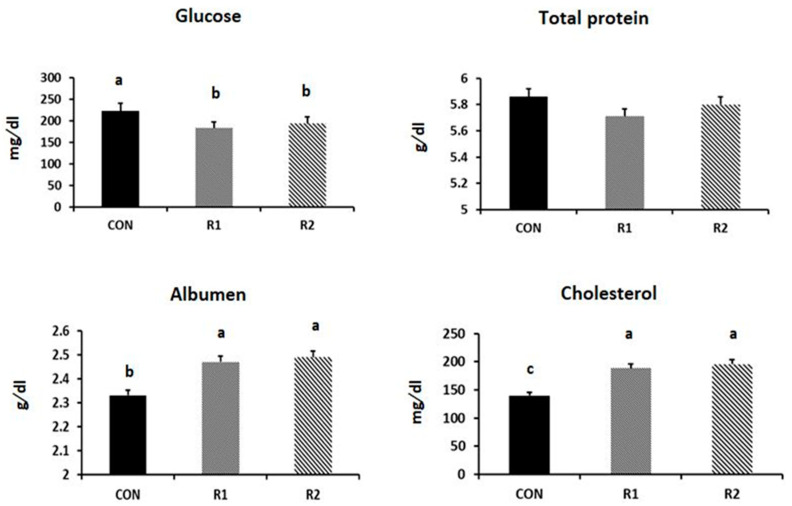

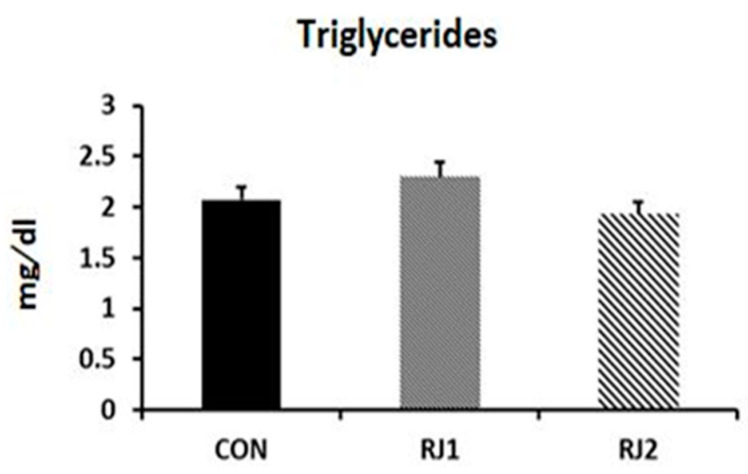
Effect of royal jelly (RJ) administration on the levels of serum glucose, total proteins, albumen, cholesterol, and triglycerides of laying hens at the late stage of production (*p* = 0.001, 0.583, 0.002, 0.018, and 0.119, respectively). ^a,b,c^ values with different superscripts differ significantly at *p* ˂ 0.05.

The administration of RJ significantly decreased the serum glucose, but increased the levels of serum albumen and cholesterol (Figure 1). On the contrary, the RJ treatment did not affect the levels of serum total proteins and triglycerides.

**Figure 2:**
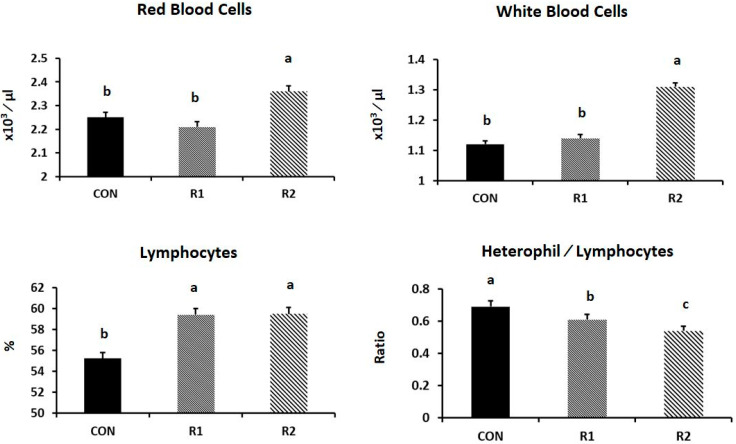
Effect of royal jelly (RJ) administration on the levels of red blood cells, white blood cells, and lymphocyte percentage, and the H/L ratio of laying hens at the late stage of production (*p* = 0.031, 0.001, 0.004, and 0.001, respectively). ^a,b,c^ values with different superscripts differ significantly at *p* ˂ 0.05.

The R1 group exhibited greater erythrocyte and total leucocyte counts (*p* = 0.031 and 0.001, respectively). Furthermore, the RJ treatment significantly increased the lymphocyte % (*p* = 0.004), but significantly reduced the H/L ratio (*p* = 0.001) (see Figure 2).

## 4. Discussion

The primary objective of this work was to investigate the impact of RJ administration on the morphology of the reproductive tract and blood parameters of aged laying hens. Several studies have been carried out regarding the applications of natural biological products to modulate the ovarian morphology of laying hens. In this context, Oguike et al. [8] reported a transformation of ovarian follicles from the resting phase to a rapid growth stage at 5–6 mm in diameter. It is also believed that the transitions occurring in the theca and granulosa tissues of the ovarian follicles may alter the follicles from non-ovulatory to ovulatory phases and a subsequent follicular maturation; however, the mechanism of delayed transition of follicles into a rapid growth phase in aged birds is not explained [8]. The current study confirmed the ameliorative action of RJ on different ovarian follicles in aged laying hens, including numbers of LYFs, SYFs, and LWFs. Further, the size and weight of the F1 follicles were significantly improved. Although the RJ mechanism of action is not yet established, it is suggested that RJ promotes follicular growth and development in a mechanism similar to that of a gonadotropin, probably due to its rich dietary components [23]. In this context, Valiollahpour et al. [24] revealed that an RJ-supplemented medium enhanced the rate of nuclear maturation in incubated oocytes. Additionally, Mazangi et al. [25] demonstrated that the enrichment of a maturation medium with RJ improved the nuclear maturation rate and minimized the apoptosis-related genes in the oocytes. Meanwhile, these positive roles are based on the fact that RJ has many protein fractions having high anti-oxidative activity and scavenging ability against active oxygen species [26]. In male rabbits, oral administration of RJ minimized the hazards of summer infertility and enhanced the physiological status of animals [27]. In vitro, other hypotheses propose that the increase in the rate of glycolysis is associated with developmental competence of oocytes. Accordingly, 90% of the total sugars found in RJ are fructose and glucose, which possibly activate the glycolysis pathway [28]. Herein, the RJ treatment significantly increased the weight of the oviduct and uterus. There are evident breakdowns in the ability of aged hens to maintain the optimum calcium level, probably due to the reduction in populations of estrogen receptors within the uterine shell gland [29]. It is also widely accepted that the fatty acids and sterols extracted from RJ have in vivo and in vitro estrogenic activities [30], similar to the action of estradiol-17 β [31]. This estrogenic activity of RJ may explain the improved morphology of the oviduct in the RJ-treated hens. Furthermore, estradiol activates the avian liver to produce the yolk precursors of yolk proteins, including vitellogenin and very-low-density lipoproteins, and consequently determines the egg mass and quality [32]. In a recent trial, Taha et al. [33] indicated that in ovo RJ injection (0.5 mL RJ/egg) improved the hatchability percentages of fertile eggs compared to the other groups. They attributed the positive effect of RJ to its enriched nutritive values, as it contains essential amino acids and vitamin precursors that enhance the growth of chick embryonic and relayed hatchability percentages. On the contrary, others have suggested that in ovo RJ injection significantly decreases hatchability compared to saline injection (Moghaddam et al. [34]).

In the current study, the RJ treatment improved the egg production rate and egg weight in aged hens, probably due to the increase in different follicular patterns (LYFs, SYFs, and LWFs) of the ovarian hierarchy. Furthermore, the higher weight and size of F1 follicles in RJ-treated birds may explain the superior egg weight. Similarly, Galal et al. [35] noticed that laying hens fed propolis-supplemented diets produced significantly heavier egg mass compared with those fed the basal diet. Additionally, Seven [36] found that the supplementation of Turkish propolis (ethanol extract, 2 and 5 g kg^−1^) improved the egg weight in Hyline hens. However, others have suggested that propolis supplements do not affect the egg production rate or quality parameters [37,38,39]. Herein, the RJ treatment improved the quality of yolk and albumen, which confirmed the previous reports [39]. Meanwhile, others noticed that propolis supplements did not affect the albumen index [36].

In this experiment, RJ treatment increased the erythrocyte counts in laying hens, suggesting a positive effect on the erythropoiesis within the bone marrow. Similarly, dietary propolis supplements increased erythrocyte counts in mice, which may be attributed to the ability of biological bee products to stimulate the proliferation and differentiation of hematopoietic cells [40]. Others have suggested that propolis may improve the digestive utilization of iron and consequently enhance the regeneration of hemoglobin [41]. Herein, the ameliorative effects of RJ on total leucocyte and lymphocyte counts agree with those reported by Orsolić and Basic [42], who stated that propolis supplements increased the leucocyte counts in mice. Taha et al. [33] also reported that in ovo RJ injection increased the lymphocyte counts in treated chickens. Meanwhile, others reported that propolis supplements had no effect on the total leucocyte counts in laying hens [43] or mice [44]. In the present study, RJ decreased the heterophil percentage and accordingly the H/L ratio, which may indicate an improved welfare condition of treated hens. Żyla et al. [45] also indicated that antioxidants and anti-free-radical supplements could improve the H/L ratio in aged laying hens. However, Çetin et al. [43] reported that propolis did not alter the differential ratios of leukocytes in laying birds. The increased serum albumen in the RJ-treated groups supports the previous findings of Kurkure et al. [46], who noticed that oral administration of RJ increased the concentration of plasma albumen in White Leghorn cockerels. Taha et al. [33] also suggested that RJ in ovo injection (0.5 mL RJ/egg) significantly increased the serum total protein, albumen, and globulin in treated chickens. In this context, the positive effect of RJ treatment on serum albumen may be attributed to its growth promotion properties and the activation of protein synthesis in liver tissues, as well as protecting body proteins from degeneration. Interestingly, the current trial reported that RJ treatment increased the serum cholesterol. Meanwhile, Taha et al. [33] demonstrated that RJ in ovo injection had a hypocholesterolaemic effect on chickens. In contrast, other researchers stated that RJ did not alter the serum biochemical indices in growing rabbits [47].

## 5. Conclusions

RJ may attenuate the negative effects of senescence in laying hens. Furthermore, RJ treatment could improve the morphology of the reproductive tract (including follicular growth and oviduct morphology), egg production rate, and the internal egg quality parameters of aged laying hens. It should be noted that the action of RJ in laying birds still needs further investigation in terms of treatment route and duration, appropriate dosage, and mechanism of action. Furthermore, the economic aspects of RJ treatment in aged laying hens should be evaluated to determine the real benefits from keeping laying hens for an additional production period.

## Figures and Tables

**Table 1:** Calculated chemical analysis of the corn-soybean diet.

Calculated Analysis	g/kg DM
^a^ ME (KJ/kg)	12,029
Crude protein	166.0
Calcium	37.7
Available phosphorus	4.5
Lysine	8.5
Leucine	12.8
Isoleucine	6.7
Arginine	9.4
Methionine	3.9
Methionine + cystiene	6.3
Tryptophan	2.2
Therionine	6.1
Phenyalanine	7.8
Histidine	4.3
Valine	7.7

^a^ ME: metabolizable energy.

**Table 2:** The chemical composition (flavonoid contents) of royal jelly (RJ).

Flavonoid Contents	^1^ TIC%	^2^ RT (min)
Pinostrobin chalcone	0.732	32.34
Pinocembrin	1.844	33.72
Tectochrysin	0.383	35.13
Chrysin	0.843	35.97
Furfuryl alcohol	0.288	2.27

^1^ TIC%: The ion current generated depends on the characteristics of the compound concerned; ^2^ RT: retention time.

**Table 3:** Effect of royal jelly (RJ) administration on the morphology of the reproductive tract of laying hens at the late stage of production.

Traits	Experimental Groups				
	^1^ CON	^2^ R_1_	^3^ R_2_	^4^ RSD	*p*-Value
^5^ LYFs	5.33 ^b^	7.43 ^a^	6.22 ^ab^	0.89	0.038
^6^ SYFs	7.38 ^b^	9.35 ^a^	9.03 ^a^	1.06	0.018
^7^ LWFs	11.32 ^b^	15.28 ^a^	14.49 ^a^	1.32	0.001
F1 diameter (mm)	29.20 ^c^	33.11 ^a^	30.94 ^b^	1.87	0.022
F1 weight (g)	8.68 ^b^	10.22 ^a^	10.31 ^a^	1.61	0.042
Oviduct weight (g)	51.56 ^b^	52.75 ^b^	55.77 ^a^	3.03	0.039
Oviduct (%)	2.73	2.71	2.82	0.18	0.084
Ovary weight (g)	33.12 ^b^	37.02 ^a^	35.49 ^a^	3.12	0.001
Ovary (%)	1.68 ^b^	1.90 ^a^	1.81 ^a^	0.17	0.028
Uterus weight (g)	21.86 ^b^	23.23 ^a,b^	25.02 ^a^	3.07	0.032
Ovarian stroma (g)	3.81	4.16	3.97	0.37	0.070

^1^ CON: control group; ^2^ R_1_; 100 mg RJ kg^−1^; ^3^ R_2_: 200 mg RJ kg^−1^; ^4^ RSD: residual standard deviation. ^5^ LYFs: number of large yellow follicles; ^6^ SYFs: number of small yellow follicles; ^7^ LWFs: number of large white follicles. a,b,c Values within a row with different superscripts differ significantly.

**Table 4:** Effect of royal jelly (RJ) administration on egg-laying performance and internal egg quality of laying hens at the late stage of production.

Traits	Experimental Groups				
	^1^ CON	^2^ R_1_	^3^ R_2_	^4^ RSD	*p*-Value
^5^ HDEP (%)	76.05 ^b^	80.36 ^a^	82.14 ^a^	1.59	0.025
Egg weight (g)	62.16 ^b^	63.34 ^ab^	66.57 ^a^	5.43	0.032
Albumen weight (g)	38.49	39.55	41.39	3.25	0.212
Albumen ratio	61.83	62.51	61.84	2.49	0.532
Yolk weight (g)	17.27 ^b^	17.94 ^b^	20.09 ^a^	1.99	0.008
Yolk ratio	28.20 ^a,b^	27.92 ^b^	29.80 ^a^	2.27	0.040
Albumen height (mm)	5.96 ^c^	7.66 ^b^	8.39 ^a^	0.61	0.001
Yolk height (mm)	15.96 ^b^	17.01 ^a^	17.56 ^a^	1.34	0.012
Yolk diameter (mm)	41.67	40.53	41.32	2.79	0.409
Haugh unit	78.31 ^b^	83.94 ^a^	85.14 ^a^	3.24	0.016
Yolk index (%)	38.47 ^b^	42.04 ^a^	42.51 ^a^	0.81	0.002

^1^ CON: control group; ^2^ R_1_; 100 mg RJ kg^−1^; ^3^ R_2_: 200 mg RJ kg^−1^; ^4^ RSD: residual standard deviation; ^5^ HDEP: hen–day egg production. a,b,c Values within a row with different superscripts differ significantly.

## Data Availability

All data generated or analyzed during this study are included in this published paper.
